# Novel miRNA Biomarkers for Patients With Duchenne Muscular Dystrophy

**DOI:** 10.3389/fneur.2022.921785

**Published:** 2022-07-06

**Authors:** Qi Meng, Jiapeng Zhang, Jingzi Zhong, Dan Zeng, Dan Lan

**Affiliations:** Department of Pediatrics, The First Affiliated Hospital of Guangxi Medical University, Nanning, China

**Keywords:** Duchenne muscular dystrophy, microRNA, myomiRs, biomarker, child, creatine kinase

## Abstract

Creatine kinase (CK) as a biomarker has long been expected to be replaced by other fluid biomarkers for Duchenne muscular dystrophy (DMD) because it is independent of disease severity. Growing evidence has demonstrated that muscle-specific microRNAs, known as myomiRs, can act as biomarkers for monitoring muscle pathology and disease severity of DMD patients. To gain insights into the relationship between serum myomiRs and clinical assessment, we measured serum levels of miR-1, miR-133a, miR-133b, miR-206, miR-208a, miR-208b, and miR-499 in 48 DMD patients by using real-time quantitative reverse transcription polymerase chain reaction. These were then compared with age, muscle strength, muscle functions, CK levels, cardiac manifestations, and mutation types (deletions, duplications, and small mutations). When compared to 53 controls, the expression levels of myomiRs were all significantly elevated (*p* < 0.05). The receiver operating characteristic curves of all seven myomiRs reflected marked differences between DMD patients and healthy controls (*p* < 0.05). We also showed that serum levels of myomiRs were positively correlated with lower limb distal muscle strength in patients of all age groups. The levels of miR-499, miR-208b, miR-133a, and miR-133b had significant negative correlations with the time to be upright from the supine position (Gowers' time) and the time taken to climb four stairs in DMD patients older than 7 years. Serum levels of miR-1, miR-133a, miR-133b, and miR-499 in patients with cardiac involvement were remarkably higher than those in non-cardiac-involved patients. There was no significant difference in levels of myomiRs between the different mutation groups. Our results indicated that serum myomiRs could be considered as novel biomarkers for monitoring pathology/pathophysiology of DMD patients. In particular, miR-499, miR-208b, miR-133a, and miR-133b might have the ability to reflect the extent of muscle impairment.

## Introduction

Duchenne muscular dystrophy (DMD) is one of the most severe progressive muscular dystrophies, and it is a lethal X-linked recessive neuromuscular disorder. It is caused by mutations in *dystrophin* gene, and the incidence is estimated at 1/3500–1/6000 live male births (1). The absence of functional dystrophin results in progressive muscle degeneration. The early manifestations of affected boys are atrophy and weakness of the proximal lower limbs and pelvic girdle muscles, pseudohypertrophy of the gastrocnemius, waddling gait, and Gowers' sign. At later stages, progressive muscle atrophy can lead to loss of ambulation between 8 and 12 years and usually death as a result of cardiac or respiratory compromise (2).

As the most common biomarker of DMD, serum creatine kinase (CK) is significantly elevated throughout the course of the disorder, although levels can gradually decline with age (3). Although serum CK is a non-specific marker for evaluating muscle pathology, its levels are not significantly associated with the severity of DMD. In addition, it can be altered by a variety of factors, including age and physical activity (4). Importantly, serum CK is also significantly elevated in many other disease states, such as severe or acute infection, acute myositis, and rhabdomyolysis. Therefore, there is a great interest to explore more reliable biomarkers of DMD to address clinical needs.

Recent research studies have shown that microRNAs (miRNAs) can be potential biomarkers of DMD. These small RNAs are a class of non-coding single-stranded RNAs with a length of approximately 22 nucleotides, which function as post-transcriptional regulators of gene expression (5). Multiple studies have shown that several miRNAs, including miR-1, miR-133a, miR-133b, miR-206, miR-208a, miR-208b, and miR-499, are enriched in striated muscles. These have been named myomiRs, and they play important roles in the differentiation of muscle and dystrophy pathology. myomiRs have been demonstrated to be elevated in the sera of DMD patients, and they are much less affected by other abnormal physiological conditions than by CK levels (6–9).

The North Star Ambulatory Assessment (NSAA) is one of the most used scales to access DMD motor ability. The higher the score, the better the motor ability of DMD patients. In the research carried out by Cacchiarelli and his co-worker in 2011, they found that serum levels of miR-1, miR-133, and miR-206 were significantly elevated in 26 DMD boys when compared to seven healthy controls. They also found that serum levels of miR-1, miR-133, and miR-206 were inversely correlated with low motor functions. Therefore, high levels of these miRNAs corresponded to low motor functions (6). However, other studies, for instance, Zaharieva et al., showed that there was no correlation between the serum levels of miR-1, miR-133a, miR-133b, miR-206, and miR-31 and NSAA scores, when they recruited 26 DMD patients (ages varied from 4 to 13 years) (7). In addition, Li and colleagues analyzed the serum levels of miR-1, miR-133, miR-206, miR-208a, miR-208b, and miR-499 in 52 DMD patients (with ages from 1 to 14 years) and 23 healthy controls. They found that all myomiRs were significantly elevated in DMD patients, and miR-499 exhibited the highest expression levels (9). Moreover, they also compared the serum levels of myomiRs with clinical information (such as age, serum CK levels, and muscle fiber composition) of 29 of their DMD patients and found that serum myomiRs could act as marker for monitoring the pathological progression of DMD (9). Although the conclusions from these research studies were different, it is certain that myomiRs can reflect certain aspects of muscle pathology.

In this study, the major goal was to use a different cohort of DMD patients and controls to further assess the ability of seven myomiRs, miR-1, miR-133a, miR-133b, miR-206, miR-208a, miR-208b, and miR-499 as potential biomarkers for DMD. We measured serum levels of these myomiRs in 48 DMD patients and compared the serum levels with age, CK levels, mutation types, cardiac manifestations, muscle strength, and muscle function to 53 control subjects. Our results showed that serum myomiRs have good diagnostic power for discriminating DMD patients from controls, and their levels in sera were significantly correlated with clinical data. In particular, miR-499, miR-208b, miR-133a, and miR-133b appeared to have the ability to act as novel biomarkers for monitoring the pathology/pathophysiology of DMD.

## Materials and Methods

### Patients

The study was approved by the Ethics Committee of the First Affiliated Hospital of Guangxi Medical University. The study adhered to the guidelines of the Declaration of Helsinki, and written informed consent was obtained from the parents or guardians of the children involved.

The children with DMD patients and the controls were of a similar age range and were recruited between January 2018 and December 2019, in the Pediatrics Department of the First Affiliated Hospital of Guangxi Medical University, China. The inclusion criteria for including children with DMD in the study were as follows: (1) genetically proven DMD, (2) no severe or moderate learning difficulties or behavioral problems, and (3) serum samples able to be collected before any treatment regimen was applied.

A total of 48 DMD patients and 53 healthy boys were included in this study. The characteristics of the participants are presented in Table 1. The mean age of DMD boys was 6.57 years (age range from 5 months to 11 years), and the mean age of the healthy controls was 5.67 years (age range between 4 and 7 years). Muscle strength was evaluated by using the Medical Research Council (MRC) scale (10). Echocardiography and 12-lead electrocardiography (ECG) were performed to evaluate the cardiac function in DMD patients with abnormal cardiac physical examinations (cardiac rhythm, heart rate abnormal). Multiplex ligation-dependent probe amplification (MLPA) and next-generation sequencing (NGS) were used to detect dystrophin gene mutations in all 48 enrolled boys. Among these cases, deletions were detected in 27 (56.25%), duplications were detected in 8 (16.67%), and small mutations were detected in 13 (27.08%) patients. All the clinical data of the DMD patients are listed in Supplementary Table 1.

**Table 1:** Characteristics of the DMD patients and controls in this study.

**Clinical characteristics**	**DMD (*n =* 48)**	**Controls (*n =* 53)**	***P*** **-value**
Age, year (Mean ± SD)	6.57 ± 2.69	5.67 ± 0.79	0.029
Sex, male	48	53	-
Without corticosteroid treatment	48	53	-
CK, U/L [median (25–75%)]	9,597.50 (6,671.00–15,177.00)	50.00 (43.50–65.00)	<0.001

*The age of the study population was normally distributed and are expressed as mean ± standard deviation (SD). Student's t-test was used for analysis. Median with 25–75% interquartile ranges was used due to the non-normal distribution of CK levels, and the Mann–Whitney test was used for analysis. A P-value < 0.05 was considered statistically significant. DMD, Duchenne muscular dystrophy; CK, creatine kinase*.

### Sample Collection

Approximately 2 mL of blood was collected from a forearm vein, and the whole blood was allowed to stand for about 1 h at room temperature before centrifugation at 3,000 r/min for 10 min at room temperature. The resultant sera were taken in a 1.5-mL enzyme-free cryotube and stored at −20°C until use. The levels of creatine kinase were analyzed by a spectrophotometric assay.

### RNA Extraction and miRNA Quantification

According to the manufacturer's instructions, small RNAs were extracted from 200 μL of the participants' serum samples by an miRcute Serum miRNA Isolation Kit (Cat. No. DP503, Tiangen, Beijing, China). Small RNAs were reverse-transcribed using the miRcute Plus miRNA First-Strand cDNA Kit (Cat. No. KR211, Tiangen, Beijing, China), which added a *poly*(A) tail to the 3′-end of miRNA. Totally, 8 μL small RNAs, 10 μL 2 × miRNA RT reaction buffer, and 2 μL miRNA RT Enzyme Mix were contained in a 20 μL reaction system of reverse transcription, and the reaction conditions were 42°C for 60 min and 95°C for 5 min. The cDNA obtained was diluted 20-fold, and 2 μL was used in a 20 μL quantitative real-time PCR reaction using the miRcute Plus miRNA qPCR Kit (cat. no. FP411, Tiangen, Beijing, China) on an ABI 7500 Real-Time PCR System (Applied Biosystems, Foster City, CA, USA). In order to improve the detection rate of low-abundance miRNAs, qPCR was performed using a program consisting of one holding stage (95°C 15 min), five pre-cycles (94 °C 20 s, 64 °C 30 s, and 72 °C 34 s), and 42 cycles (94 °C 20 s and 60°C 34 s). We used external control for miRNA (cat. no. CR100-1, Tiangen, Beijing, China) to normalize expression data. The forward primers for external control, miR-1, miR-133a, miR-133b, miR-206, miR-208a, miR-208b, and miR-499 were purchased from Tiangen Biotechnology Co., Ltd., China (Supplementary Table 2). Universal reverse primers for qPCR were provided in a detection kit. The relative expression levels of miRNAs were calculated using the 2^−ΔΔCt^ method.

### Statistical Analysis

Before statistical evaluation, data were tested for normality by using the Shapiro–Wilk test. Normally distributed data were presented as mean ± standard deviation (SD), whereas skewed variables were described as medians with 25% to 75% interquartile range (IQR). For comparison between two groups, statistical differences were analyzed by using either Student's *t*-test or the Mann–Whitney *U*-test depending on whether the data were normally distributed. The Kruskal–Wallis test was performed to confirm the differences of multiple group comparisons. Receiver operating characteristic (ROC) curves were plotted for the diagnostic analysis of the profiles obtained. The area under the ROC curve (AUC) was calculated to evaluate the discrimination accuracy. Correlations between variables were calculated using the Spearman correlation coefficient. IBM SPSS 24.0 (SPSS Inc. USA) and GraphPad 8.3 software (GraphPad Software Inc. USA) packages were used for all the statistical analyses. All *p-*values of < 0.05 were considered significant.

## Results

### Serum Levels of myomiRs Were Significantly Elevated in DMD Patients

**Figure 1:**
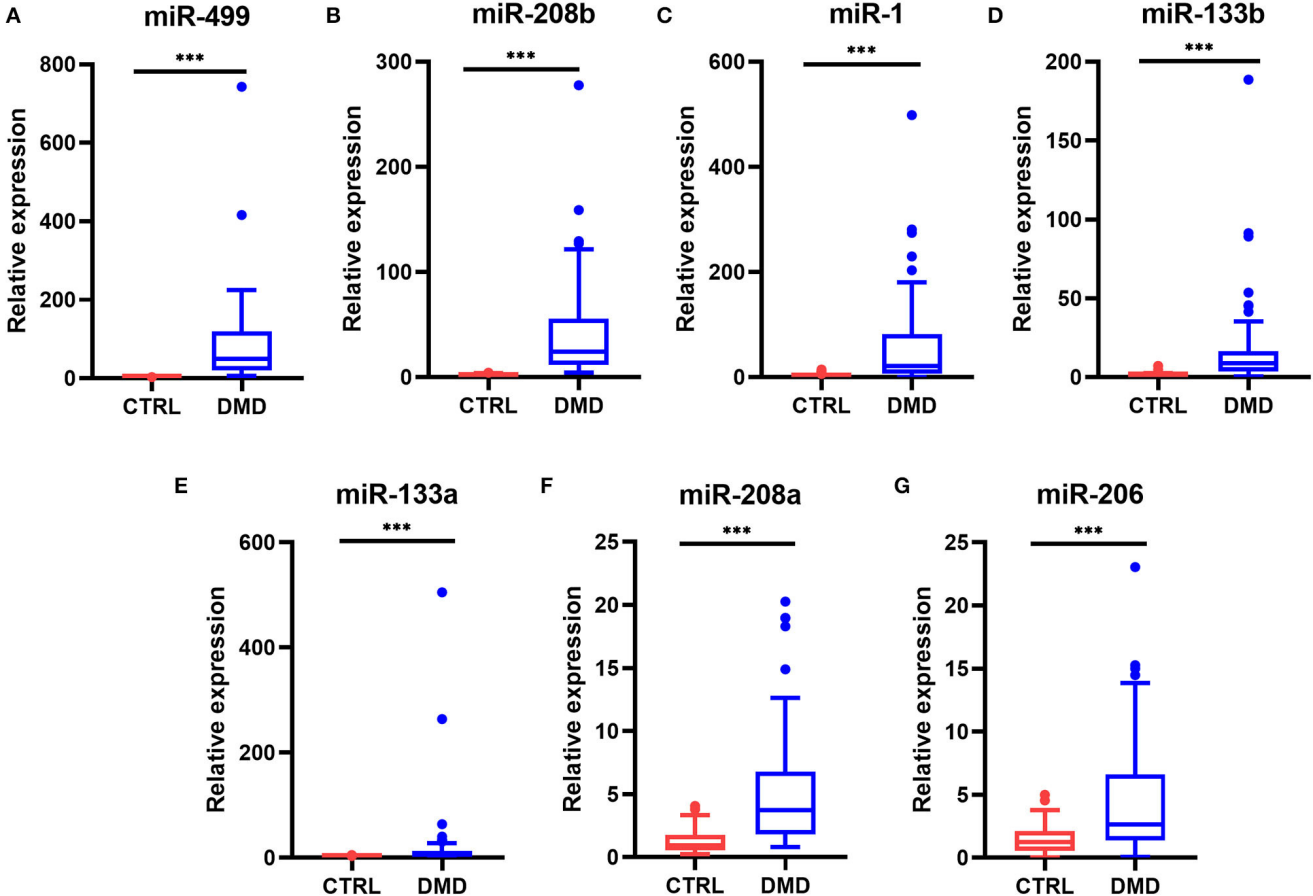
Levels of myomiRs in the serum of DMD patients compared to controls. Serum levels of miR-499 **(A)**, miR-208b **(B)**, miR-1 **(C)**, miR-133b **(D)**, miR-133a **(E)**, miR-208a **(F)**, and miR-206 **(G)** in 53 healthy controls (red) and 48 DMD patients (blue) are shown as Tukey box plots. The lines within the boxes indicate the median levels, the box limits indicate first and third quartiles, the highest line means the Q3 + 1.5 IQR, lowest line means Q1 – 1.5 IQR, and individual points represent outliers. As data of both the DMD and control group were non-normally distributed variables, the non-parametric Mann–Whitney U-test was used for comparison between the two groups. All *p-*values were two-tailed, and *p* < 0.05 was considered statistically significant. ^***^p < 0.0001. DMD, Duchenne muscular dystrophy; CTRL, control.

Real-time PCR was used to quantify the levels of miR-1, miR-133a, miR-133b, miR-206, miR-208a, miR-208b, and miR-499 in the sera of healthy controls (*n* = 53) and DMD patients (*n* = 48). Serum levels of myomiRs were found to be significantly elevated in the DMD group compared to controls (Figure 1). The relative expression levels of myomiRs are listed in Table 2. miR-499 had the highest expression level, which was 45.8 times higher than of the control group. This was followed by miR-208b (*p* < 0.0001, 26.23-fold), miR-1 (*p* < 0.0001, 22.13-fold), miR-133b (*p* < 0.0001, 8.64-fold), miR-133a (*p* < 0.0001, 5.71-fold), miR-208a (*p* < 0.0001, 4.00-fold), and miR-206 (*p* < 0.0001, 2.13-fold).

**Table 2:** Serum levels of myomiRs in DMD patients vs. controls.

**miRNA levels (2^**−*ΔΔCT***^)**	**DMD**	**Controls**	***P*** **-value**
**[median (25–75%)]**	**(*n =* 48)**	**(*n =* 53)**	
miR-499	49.47 (20.16–118.82)	1.08 (0.74–1.51)	<0.0001
miR-208b	24.46 (11.52–55.53)	0.94 (0.56–1.93)	<0.0001
miR-1	21.47 (6.39–81.65)	0.97 (0.47–2.10)	<0.0001
miR-133b	8.64 (3.68–16.59)	1.00 (0.67–1.62)	<0.0001
miR-133a	6.85 (1.93–12.91)	1.20 (0.59–1.69)	<0.0001
miR-208a	3.72 (1.81–6.78)	0.93 (0.56–1.78)	<0.0001
miR-206	2.66 (1.39–6.58)	1.25 (0.53–2.11)	<0.0001

*The serum levels of myomiRs in DMD and control groups were not normally distributed and are expressed as median and 25–75 percentiles. The Mann–Whitney test was used to perform the statistical analysis. A P-value < 0.05 was considered statistically significant. DMD, Duchenne muscular dystrophy*.

**Figure 2:**
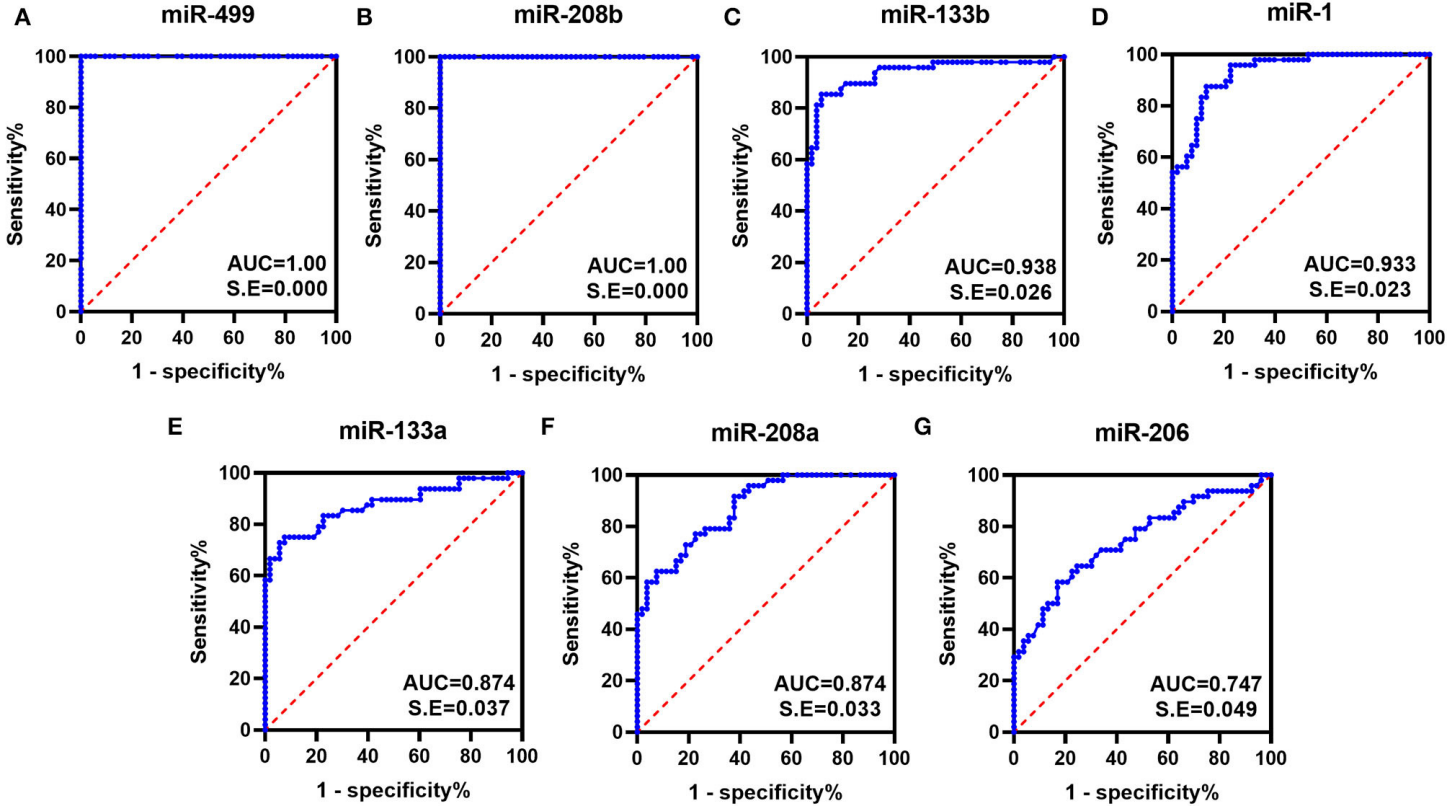
ROC curves obtained for the myomiRs. ROC curves were plotted to evaluate the diagnostic performance of miR-499 **(A)**, miR-208b **(B)**, miR-133b **(C)**, miR-1 **(D)**, miR-133a **(E)**, miR-208a **(F)**, and miR-206 **(G)**. The AUCs were calculated for the measurement of discrimination accuracy. All had *p-*values < 0.05.

To evaluate the diagnostic performance of myomiRs, the ROC curves were drawn, using the AUCs to summarize the sensitivity and specificity. Generally, the value of AUC is between 0.5 and 1.0, and the larger the area the better diagnostic effectiveness. An AUC >0.7 indicates good diagnostic effectiveness, and when it is >0.9, it represents an extremely good effect. As shown in Figure 2, the AUCs for all the miRNAs were >0.747, which was observed for miR-206. miR-499 and miR-208b showed a perfect statistical value (AUC of 1.0 in both cases), and miR-133b, miR-1, miR-208a, and miR-133a also displayed very good diagnostic power in distinguishing DMD patients from controls subjects (AUCs of 0.938, 0.933, 0.874, and 0.874, respectively).

### Correlation Between Serum myomiR Levels and Age

In the patients with DMD, there was an increase in muscle damage with age, and muscle strength and function tend to show a downward trend. To explore whether serum myomiR levels would change with age, we divided the DMD patients into different age groups (≤2, 2–7, >7 years, and all ages) according to the progression of the disease (2) and analyzed the correlations between miRNA levels and age. As shown in Table 3, there was no statistical significance observed in the all age groups. Notably, in further sub-groups, miR-208b showed a positive correlation with age in 2- to 7-year-olds, and miR-133b was positively correlated with age in >7-year-olds. We supposed it is a good signal that different ages showed variations in sensitivity to some miRNAs. Furthermore, we also analyzed the correlation between serum CK levels and age. Consistent with previous research, CK levels were not significantly correlated with age (Table 3).

**Table 3:** Correlation between serum myomiR levels and age.

**Age**	**≤2 years old (*****n =*** **3)**		**2–7 years old (*****n =*** **23)**		**>7 years old (*****n =*** **22)**		**All ages (*****n =*** **48)**	
	***r***	***p***	***r***	***p***	***r***	***p***	***r***	***p***
miR-1	1	0.3333	0.3238	0.1318	0.2181	0.3296	−0.0203	0.8910
miR-133a	0.5	>0.9999	0.3426	0.1096	0.4181	0.0528	0.0859	0.5616
miR-133b	−0.5	>0.9999	0.3658	0.0860	0.4576	0.0322	0.1411	0.3388
miR-206	0.5	>0.9999	0.3055	0.1563	0.3661	0.0938	0.05230	0.7207
miR-208a	0.5	>0.9999	0.3629	0.0888	0.1842	0.4118	−0.0015	0.9921
miR-208b	0.5	>0.9999	0.4861	0.0187	0.1322	0.5576	0.0144	0.9224
miR-499	1	0.3333	0.3926	0.0639	−0.05085	0.8222	−0.0588	0.6917
CK	0.5	>0.9999	−0.1035	0.6385	−0.3328	0.1302	−0.0775	0.6007

*Spearman's correlation coefficient was used to analyze the correlation between the serum myomiR levels and age of DMD patients since the data were not normally distributed. A P-value < 0.05 was considered statistically significant*.

### The Correlation Between Serum Levels of myomiRs and Clinical Indicators

To determine whether myomiRs were correlated with the degree to which the muscles were impaired, we collected several clinical indicators to evaluate disease progression in DMD patients (Table 4). These were correlated with serum levels of miRNAs by using Spearman's rank correlation. A 10-point scale grading system of muscle strength was used to score the upper and lower limb muscle strengths of DMD patients. The time they took to walk 10 meters, to climb four stairs, and to be upright from the supine position (Gowers' time) were used to test muscle functions (8, 11). Because of some patients' non-collaboration, non-ambulation, or young age, only 37 DMD boys and 27 DMD boys had completed the tests for muscle strength and function, respectively.

**Table 4:** Results of the clinical evaluations of children with Duchenne muscular dystrophy.

**Clinical evaluations (Mean ±SD)**	***n***	**Results of tests**
Upper limb proximal muscle strength (score)	37	7.784 ± 1.377
Lower limb proximal muscle strength (score)	37	7.405 ± 1.423
Lower limb distal muscle strength (score)	37	7.514 ± 1.465
Time to walk 10 meters (second)	27	6.62 ± 2.51
Time to climb 4 stairs (second)	27	5.81 ± 4.24
Gowers' time (second)	27	7.20 ± 3.94

*The data in the table follows normal distribution and are expressed as mean ± standard deviation (SD). Gowers' time: the time taken to change from the supine position to upright*.

Tables 5, 6 consist of a summary of the correlation of miRNAs and CK levels together with muscle strength and function, respectively. The results showed that CK levels had no significant correlations with muscle strength and function, which is consistent with a previous study (8). All the levels of miRNAs had positive correlations with lower limb distal muscle strength in all age groups (miR-1, *r* = 0.41; miR-133a, *r* = 0.61; miR-133b, *r* = 0.61; miR-206, *r* = 0.44; miR-208a, *r* = 0.39; miR-208b, *r* = 0.45; miR-499, *r* = 0.51; *p* < 0.05 in all cases; Table 5). Moreover, the levels of miR-133a, miR-133b, and miR-499 had significant positive correlations with lower limb proximal muscle strength (*r* = 0.42, 0.41, and 0.34, respectively; Table 5). It is noteworthy that miR-499, miR-133a, and miR-133b were significantly and positively correlated with lower limb distal muscle strength in the groups with 2- to 7-year-olds and >7-year-olds. These results indicated that the stronger the muscle strength, the higher the expression of myomiRs.

**Table 5:** Correlation between serum myomiRs and CK levels and muscle strength.

**Muscle strength**	**≤2 years old (*****n =*** **0)**		**2–7 years old (*****n =*** **16)**		**>7 years old (*****n =*** **21)**		**All ages (*****n =*** **37)**	
**Upper limb proximal**	***r***	***p*** **-value**	***r***	***p*** **-value**	***r***	***p*** **-value**	***r***	***p*** **-value**
miR-1	-	-	0.2796	0.2914	−0.0965	0.6772	0.0919	0.5884
miR-133a	-	-	0.4118	0.1136	0.2135	0.3528	0.2933	0.0781
miR-133b	-	-	0.4434	0.0867	0.1797	0.4358	0.2840	0.0884
miR-206	-	-	0.3803	0.1460	−0.0514	0.8248	0.1486	0.3802
miR-208a	-	-	0.1263	0.6384	−0.0892	0.7007	0.0272	0.8732
miR-208b	-	-	0.2781	0.2941	−0.1642	0.4770	0.0633	0.7098
miR-499	-	-	0.3367	0.2008	0.0303	0.8963	0.2001	0.2351
CK	-	-	0.2029	0.4472	−0.2093	0.3626	0.0326	0.8482
**Lower limb proximal**	***r***	***p*** **-value**	***r***	***p*** **-value**	***r***	***p*** **-value**	***r***	***p*** **-value**
miR-1	-	-	0.2628	0.3223	0.1110	0.6320	0.2676	0.1093
miR-133a	-	-	0.3636	0.1655	0.4148	0.0615	0.4198	0.0097
miR-133b	-	-	0.4446	0.0857	0.4229	0.0561	0.4070	0.0124
miR-206	-	-	0.2888	0.2754	0.1523	0.5100	0.2273	0.1760
miR-208a	-	-	0.1895	0.4786	0.0542	0.8157	0.1612	0.3405
miR-208b	-	-	0.3025	0.2525	0.0717	0.7573	0.2299	0.1710
miR-499	-	-	0.3254	0.2169	0.2741	0.2293	0.3429	0.0377
CK	-	-	0.4033	0.1217	−0.0081	0.9721	0.2121	0.2076
**Lower limb distal**	***r***	***p*** **-value**	***r***	***p*** **-value**	***r***	***p*** **-value**	***r***	***p*** **-value**
miR-1	-	-	0.3529	0.1793	0.4062	0.0677	0.4103	0.0117
miR-133a	-	-	0.5470	0.0304	0.6766	0.0008	0.6101	<0.0001
miR-133b	-	-	0.5913	0.0177	0.6973	0.0004	0.6112	<0.0001
miR-206	-	-	0.4599	0.0746	0.4156	0.0610	0.4353	0.0071
miR-208a	-	-	0.3499	0.1833	0.3581	0.1109	0.3864	0.0182
miR-208b	-	-	0.4981	0.0515	0.3841	0.0856	0.4486	0.0054
miR-499	-	-	0.5286	0.0373	0.4410	0.0454	0.5097	0.0013
CK	-	-	0.4904	0.0556	0.1245	0.5909	0.3222	0.0518

*Spearman's correlation coefficient was used since the data were not normally distributed. A P-value < 0.05 was considered statistically significant. CK, creatine kinase*.

**Table 6:** Correlation between serum myomiRs and CK levels and muscle functions.

**Muscle function**	**≤2 years old (*****n =*** **0)**		**2–7 years old (*****n =*** **13)**		**>7 years old (*****n =*** **14)**		**All ages (*****n =*** **27)**	
**Time to walk 10m**	***r***	***p*** **-value**	***r***	***p*** **-value**	***r***	***p*** **-value**	***r***	***p*** **-value**
miR-1	-	-	0.0495	0.8735	−0.4769	0.0872	−0.2644	0.1826
miR-133a	-	-	−0.0138	0.9676	−0.5253	0.0567	−0.3212	0.1023
miR-133b	-	-	−0.0660	0.8308	−0.6176	0.0212	−0.3591	0.0658
miR-206	-	-	−0.0468	0.8811	−0.0857	0.7732	−0.0826	0.6821
miR-208a	-	-	0.0055	0.9889	−0.0550	0.8557	−0.0791	0.6950
miR-208b	-	-	−0.1706	0.5746	−0.4110	0.1458	−0.3542	0.0699
miR-499	-	-	−0.1320	0.6652	−0.5165	0.0615	−0.3841	0.0479
CK	-	-	−0.2091	0.4897	−0.1736	0.5526	−0.2534	0.2021
**Time to climb 4 stairs**	***r***	***p*** **-value**	***r***	***p*** **-value**	***r***	***p*** **-value**	***r***	***p*** **-value**
miR-1	-	-	0.2143	0.4819	−0.6000	0.0261	−0.2125	0.2873
miR-133a	-	-	−0.0440	0.8917	−0.6571	0.0128	−0.3740	0.0546
miR-133b	-	-	−0.0604	0.8491	−0.7187	0.005	−0.4241	0.0275
miR-206	-	-	−0.2088	0.4934	−0.2527	0.3825	−0.1881	0.3475
miR-208a	-	-	0.0165	0.9639	−0.1824	0.5321	−0.1172	0.5603
miR-208b	-	-	−0.1978	0.5171	−0.5912	0.0288	−0.4060	0.0356
miR-499	-	-	−0.0604	0.8491	−0.5516	0.0438	−0.3914	0.0435
CK	-	-	−0.0440	0.8917	−0.1341	0.6485	−0.1053	0.6011
**Gowers**' **time**	***r***	***p*** **-value**	***r***	***p*** **-value**	***r***	***p*** **-value**	***r***	***p*** **-value**
miR-1	-	-	0.0440	0.8917	−0.4813	0.0840	−0.2216	0.2666
miR-133a	-	-	−0.2637	0.3835	−0.7363	0.0037	−0.4701	0.0134
miR-133b	-	-	−0.2637	0.3835	−0.8198	0.0006	−0.5353	0.0040
miR-206	-	-	−0.4615	0.1150	−0.3451	0.2272	−0.3590	0.0659
miR-208a	-	-	−0.2198	0.4703	−0.2923	0.3100	−0.2900	0.1423
miR-208b	-	-	−0.3681	0.2167	−0.6264	0.0191	−0.4676	0.0139
miR-499	-	-	−0.1374	0.6561	−0.5780	0.0333	−0.4011	0.0381
CK	-	-	−0.0769	0.8064	−0.3363	0.2399	−0.2680	0.1765

*Spearman's correlation coefficient was used since the data were not normally distributed. A P-value < 0.05 was considered statistically significant. CK, creatine kinase. Gowers' time: the time taken to change from the supine position to upright*.

As shown in Table 6, when analyzing the correlations between serum levels of miRNAs, significant negative correlations were seen in the all age groups, especially in the case of miR-499 for the time to walk 10 m (miR-499, *r* = −0.38), the time to climbing four stairs (miR-499, *r* = −0.39; miR-133b, *r* = −0.42; miR-208b, *r* = −0.41), and Gowers' time (miR-499, *r* = −0.40; miR-133a, *r* = −0.47; miR-133b, *r* = −0.54; miR-208b, *r* = −0.47) (*p* < 0.05 in all cases). In addition, we observed that miR-499, miR-208b, miR-133a, and miR-133b were negatively correlated with the time to walk 10 m and Gowers' time in DMD patients who were older than 7 years (Table 6). These observations suggested that serum myomiRs were able to reflect the physiological muscle state of DMD patients. In particular, miR-499, miR-208b, miR-133a, and miR-133b showed a great potential for monitoring the progression of DMD in patients.

### Serum Levels of myomiRs in DMD Patients With Cardiac Involvement

**Figure 3:**
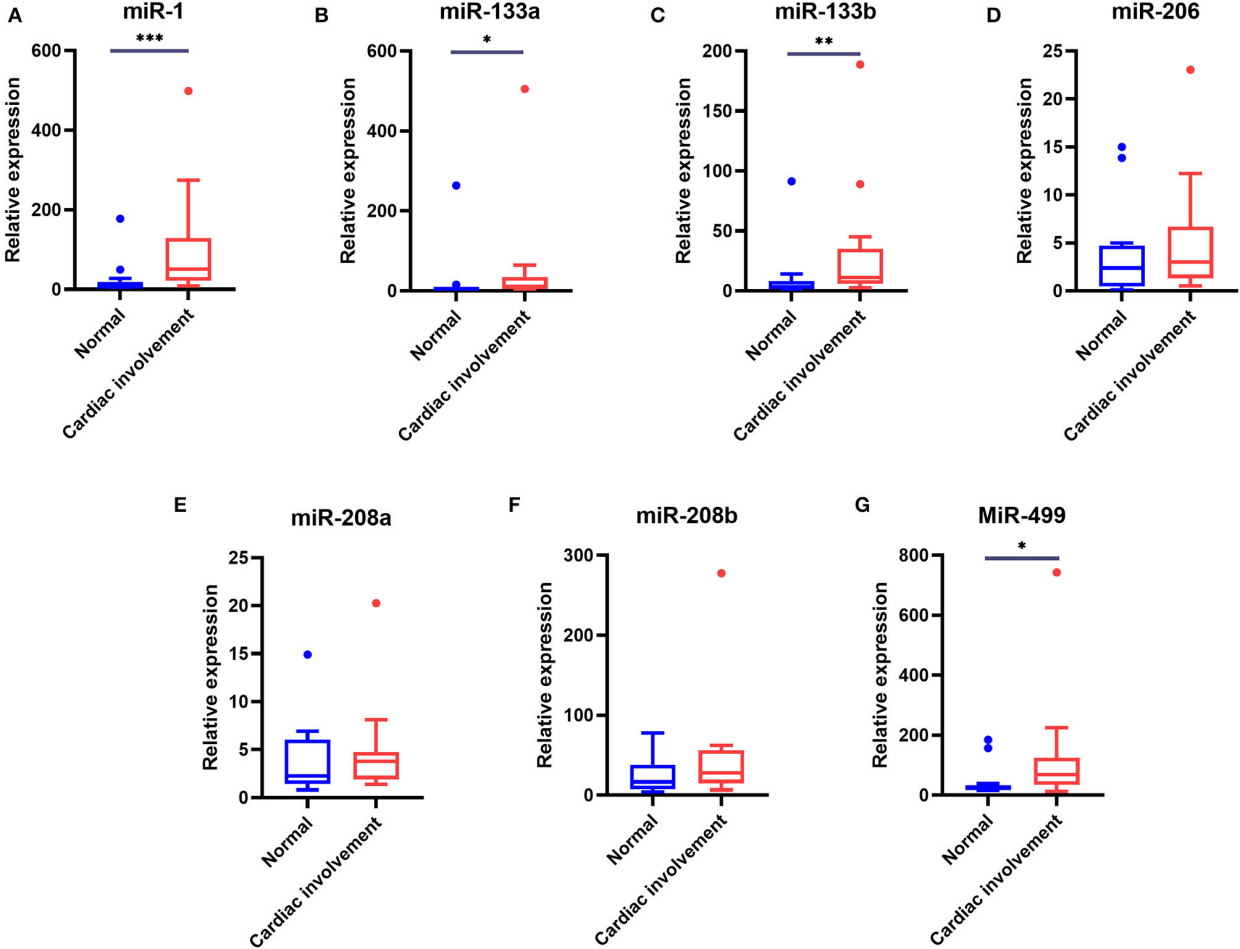
Comparison of the serum levels of myomiRs in DMD patients with cardiac involvement and normal groups. Of the patients who underwent cardiac function evaluation, 13 patients with normal ECG and echocardiography were defined as the normal group, 15 patients with one or more abnormalities were defined as the cardiac involvement group. Tukey box plots were drawn to represent the serum levels of myomiRs, miR-1 **(A)**, miR-133a **(B)**, miR-133b **(C)**, miR-206 **(D)**, miR-208a **(E)**, miR-208b **(F)**, and miR-499 **(G)**, in normal and abnormal groups. The lines in the boxes indicate the median levels, the box limits indicate first and third quartiles, the highest line means Q3+1.5 IQR, lowest line means Q1-1.5 IQR, and individual points represent outliers. As data of the two groups were non-normally distributed variables, the non-parametric Mann–Whitney *U*-test was used for comparison between the two groups. *p < 0.05, **p = 0.001–0.01, ***p < 0.0001. *P* < 0.05 was considered statistically significant.

Of the 48 DMD patients, 31 underwent echocardiography and 28 underwent 12-lead ECG. These patients were then grouped according to whether they had cardiac changes (enlarged left ventricle, sinus arrhythmia, heart block, etc.,) (12): normal group: neither echocardiography nor ECG displayed any abnormality, 13 patients; cardiac involvement group: either echocardiography or ECG abnormality, or both, 15 patients. We next analyzed the miRNA levels in these two groups. As shown in Table 7 and Figure 3, the serum levels of miR-1, miR-133a, miR-133b, and miR-499 in the cardiac involvement group were significantly higher than those in the normal group.

**Table 7:** Comparison of serum miRNA levels in DMD patients with cardiac involvement or non-cardiac-involved patients.

**miRNA level**	**Normal cardiac**	**Cardiac involvement**	***p*** **-value**
**[median (25%-75%)]**	**(*n =* 13)**	**(*n =* 15)**	
miR-1	6.93 (2.43–18.57)	50.74 (21.53–129.40)	0.0006
miR-133a	1.84 (1.19–5.48)	10.58 (5.13–34.78)	0.0148
miR-133b	3.32 (1.50–8.27)	11.20 (5.84–35.18)	0.0037
miR-206	2.39 (0.48–4.75)	3.01 (1.32–6.7)	0.3936
miR-208a	2.23 (1.42–6.03)	3.75 (1.90–4.75)	0.4745
miR-208b	16.59 (7.63–37.85)	27.78 (14.99–56.07)	0.1846
miR-499	24.04 (16.55–33.42)	68.79 (34.59–124.90)	0.0367

*The serum levels of myomiRs in cardiac involvement group and normal group were not normally distributed and are expressed as median and 25–75 percentiles. The Mann–Whitney test was used to perform the statistical analysis. A P-value < 0.05 was considered statistically significant. DMD, Duchenne muscular dystrophy*.

### Relationship Between Serum Levels and Different Types of Gene Mutations

**Figure 4:**
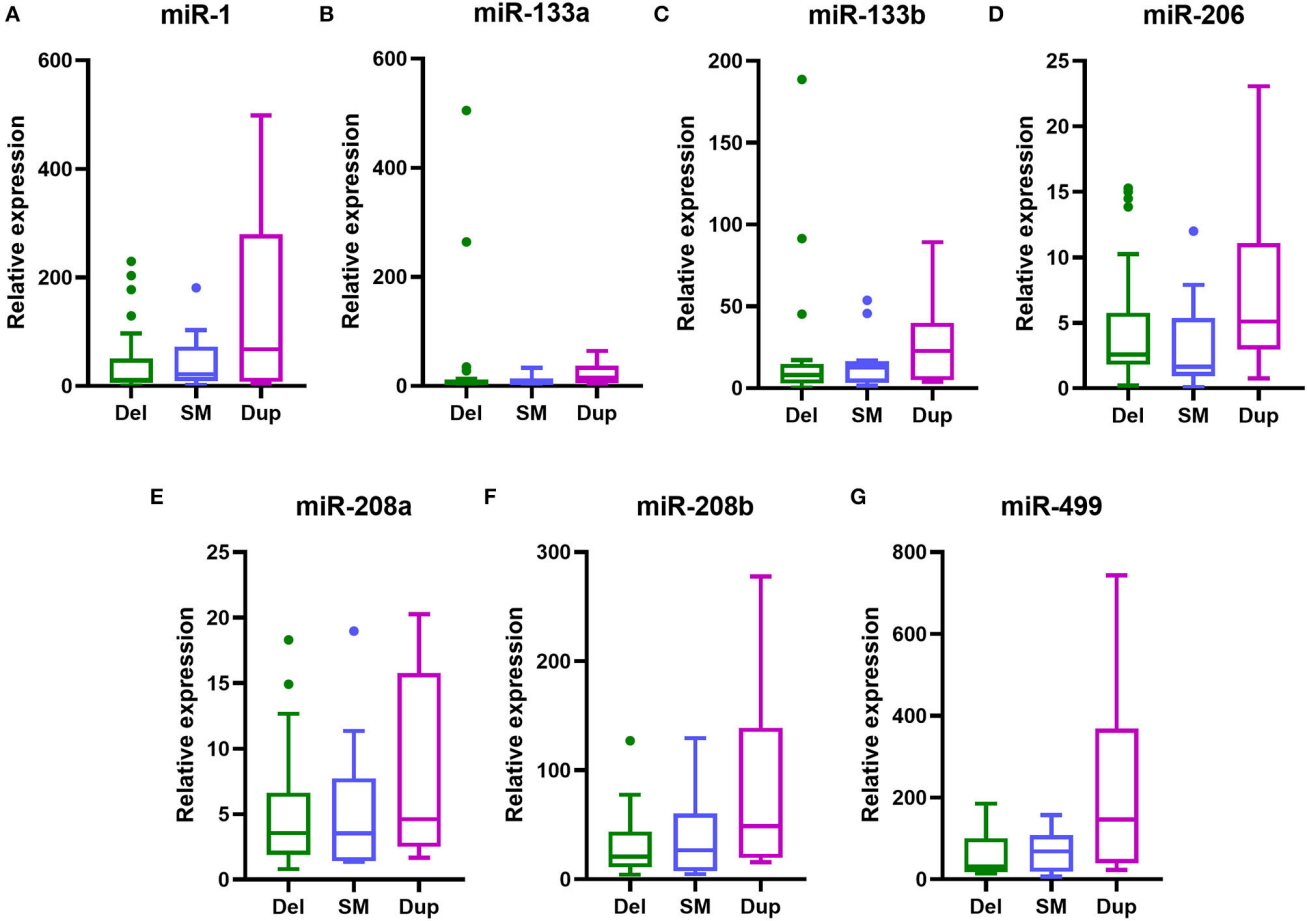
Serum levels of myomiRs with different genotypes of DMD mutations. Serum levels of myomiRs, **(A)** miR-1, **(B)** miR-133a, **(C)** miR-133b, **(D)** miR-206, **(E)** miR-208a, **(F)** miR-208b and **(G)** miR-499, of the 48 DMD patients divided into three groups (green for Del, *n* = 27; light blue for SM, *n* = 13; purple for Dup, *n* = 8) are shown as Tukey box plots. The lines in the boxes indicate the median levels, the box limits indicate first and third quartiles, the highest line means the Q3 + 1.5 IQR, lowest line means Q1 – 1.5 IQR, and individual points represent outliers. As data of the three groups were non-normally distributed variables, the non-parametric Kruskal–Wallis test was used for multiple comparison. Del, deletion; SM, small mutation; Dup, duplication.

To investigate the connections between serum levels of miRNAs and mutation types of DMD, we divided 48 DMD patients into three groups according to mutation types: deletions, duplications, and small mutations. Then we analyzed the miRNA levels of each group, and inter-group comparisons were carried out using the Kruskal–Wallis test. The results are shown in Table 8 and Figure 4. Although the median levels of seven miRNAs in the duplication group were higher than those in the other groups, the differences were not statistically significant. This may, in part, be due to the small numbers in each group.

**Table 8:** Comparison of serum miRNA levels in DMD patients with different gene mutations.

**miRNA level**	**Deletion mutation**	**Small mutation**	**Duplication mutation**	***p*** **-value**
**[median (25–75%)]**	**(*n =* 27)**	**(*n =* 13)**	**(*n =* 8)**	
miR-1	11.20 (5.55–50.74)	21.53 (9.27–72.33)	67.63 (7.80–279.50)	0.2403
miR-133a	5.58 (1.84–11.63)	8.03 (1.69–14.21)	15.48 (4.49–37.22)	0.3215
miR-133b	7.93 (2.76–14.69)	12.70 (3.39–16.43)	22.84 (4.91–39.91)	0.2650
miR-206	2.58 (1.81–5.76)	1.65 (0.95–5.37)	5.11 (2.97–11.09)	0.1246
miR-208a	3.56 (1.90–6.64)	3.54 (1.40–7.75)	4.61 (2.49–15.76)	0.5250
miR-208b	20.76 (11.13–43.64)	26.71 (7.45–60.47)	48.76 (19.57–139.00)	0.1533
miR-499	31.72 (17.52–100.70)	68.79 (18.62–108.10)	146.80 (39.95–368.40)	0.0585

*The serum levels of myomiRs in deletion mutation, small mutation, and duplication mutation groups were not normally distributed and are expressed as median and 25–75 percentiles. The Kruskal–Wallis test was used to perform the statistical analysis. A P-value < 0.05 was considered statistically significant. DMD, Duchenne muscular dystrophy*.

## Discussion

In the past decade, miRNAs had been demonstrated to be reliable biomarkers for diseases including cancer, heart failure, and leukemia (13). It is well established that miRNAs play important roles in the mechanisms of muscle growth and development, and several miRNAs, called myomiRs, have been shown to be specifically expressed in muscle cells (14). The serum CK level is the most commonly used biomarker for the detection of DMD, but since CK is relatively limited in evaluating disease severity, it is necessary to search for novel reliable biomarkers. In our research, we examined the serum levels of myomiRs to evaluate their potential for monitoring the disease progress of DMD.

In this study, we confirmed that serum levels of myomiRs, especially miR-499 and miR-208b, were significantly elevated in DMD children. Moreover, ROC curve analysis showed that all the myomiRs studied were capable of discriminating the presence of DMD from healthy children. It is interesting to note that the levels of miRNA were not the same. It is up to 45.8-fold with respect to miR-499 and only 2.13-fold for miR-206. We believe the differential expression of miRNAs is caused by a variety of factors. Cacchiarelli et al. (15) found that after receiving exon skipping treatment, the levels of miR-1, miR-133a, miR-29c, miR-30c, and miR-206 in mdx mice increased when the amount of dystrophin in muscles was restored. This indicated that the expression levels of some myomiRs in muscles are related to the amounts of dystrophin. After further research, they suggested that the differences in serum levels of miRNAs resulted from the intensive degeneration occurring in muscles during DMD (6).

However, incongruence expression levels of miRNA between muscles and serum were observed in a later study, and this indicated that the secretion of miRNA may be selective and affected by several factors (16). Then Li's group found that TNF-α, TGF-β, and FGF could stimulate a mouse muscle myoblast cell line, C2C12, to secrete certain myomiRs under differentiation culture conditions (9). Based on the fact that cytokines such as TNF-α increase with age, they speculated that the increase in serum myomiRs was due to the differentiation of muscle cells (9). In addition, TGF-β increased the levels of miR-1, miR-133, miR-206, and miR-208b but did not influence the levels of miR-499 (9). This also suggested that the differential expression of myomiRs can be affected by various factors.

In 2016, Coenen-Stass noted that the abundance of serum muscle-specific miRNAs may be a function of muscle mass and growth, the degenerative/regenerative status, and tissue expression levels (17). His research showed that myomiRs (miR-1, miR-133a, and miR-206) were secreted during myogenic differentiation, and the decline in muscle mass leads to decreased muscle-specific miRNA secretion (17). Therefore, we can speculate that the expression of myomiRs decreased progressively with the progressive muscle atrophy in DMD patients. Recent research showed that dystrophic mice with skewed X-chromosome inactivation expressed varying levels of dystrophin protein, which correlated with different serum miRNA levels (18). Moreover, Coenen-Stass et al. (19) observed different rates of degradation of miR-1, miR-133a, miR-206, miR-16, miR-223, and let-7a in the serum of mdx mice under simulated physiological conditions. miR-1 was the fastest, and miR-133a was the slowest (19). They concluded that miRNAs were prone to degradation based on their sequences (19). Hence, we speculate that one of the reasons for the differential expression of miRNA is that the nodes we observed were in different degradation states.

Previous studies showed that the serum levels of miRNAs were correlated with clinical assessments of patients. Zahariaeva compared the serum levels of miR-1, miR-133a, miR-133b, miR-206, and miR-31 in 26 ambulant DMD patients and 18 non-ambulant DMD patients (7). The former had significantly higher levels than the latter, indicating that the serum levels of myomiRs were related to muscle functions. We also correlated serum myomiRs and CK levels with muscle strength and function in different age groups. As previously reported (8), we did not observe any correlation between CK levels and muscle strength or functions, and it could not monitor the disease progression of DMD patients.

We did find that serum levels of miR-133a, miR-133b, and miR-499 were positively correlated with muscle strength in 2- to 7- and >7-year-old DMD patients. Moreover, we observed miR-133a, miR-133b, miR-208b, and miR-499 had negative correlations with the time to walk 10 m and Gowers' time in DMD patients older than 7 years, respectively. Our data showed that the higher the levels of myomiRs, the stronger the muscle strength. This is consistent with our hypothesis that myomiRs decreased with progressive muscle atrophy in DMD patients. However, in an earlier research study, there was an inverse correlation between serum levels of miR-1, miR-133, and miR-206 and the NSAA scores in 10-, 3- to 6-year-old DMD boys (6). This meant that a higher level of miRNAs corresponded to a worse motor function. Hu et al. also supported this conclusion (8). We believe that such contradictory conclusions may be due to the small number of patients in these studies. In addition, because the patients' muscle involvement is different, the pathological states of the muscle fibers would be different, as noted in other studies (9). Plasma miR-133b was reported to increase in the fast-twitch extensor digitorum longus (EDL) compared to the slow-twitch soleus (SOL) in damaged muscles, and levels of miR-206 were only elevated in SOL (20). miR-206, miR-208b, and miR-499 were abundant in slow-twitch fibers, which are influenced subsequent to fast-twitch fibers during DMD (21).

Based upon our results, there was a non-negligible observation that the patients' muscle strength was proportional to serum miRNA levels, while these were low in healthy controls. This may be related to the pathological changes in the muscles of the patients. In the early stage of DMD, muscle degeneration, regeneration, and fiber size variations occur. As the disease stage progresses, muscle fibers gradually reduce due to the decrease in muscle regeneration, while they are replaced by connective and adipose tissues (22).The increase in serum miRNAs may be due to the damage of muscle membranes, which can leak miRNAs leaking into the circulation.

We also analyzed the relationship between serum myomiR levels and age. Although there was no correlation between miRNAs and age in the all age groups, miR-208b and miR-133b were positively correlated with age in the 2- to 7- and >7-year-old groups, respectively. In DMD patients older than 7 years, the accelerated impairment of muscles led to a decline in muscle strength and functions (2). Considering the possibility that different muscle fibers are affected at different ages, we recommend the use of distinct miRNAs for monitoring pathological progress at various ages.

We also showed that serum miR-499 was positively correlated with lower limb muscle strength; thus, miR-499 can predict muscle pathology because the lower limbs are preferentially affected in DMD. Moreover, miR-499 showed a significant negative correlation with muscle functions, although the number of patients studied was small. The relevance of muscle-specific miRNAs and clinical assessment still needs further validation.

Muscle atrophy is a major feature of many myopathies, including DMD, Becker muscular dystrophy (BMD), limb-girdle muscle dystrophy, myotonic dystrophy, amyotrophic lateral sclerosis, and spinal muscular atrophy. Circulating myomiRs have also been demonstrated to be dysregulated in these myopathies and could potentially be used as biomarkers for these myopathies (23–30). Wang et al. reported that after 15 healthy adult male individuals accepted to the head-down bed rest experiment for 45 days, muscle atrophy occurred. Serum miR-23a, miR-206, and miR-499 were significantly elevated, and these were positively correlated with the soleus volume loss (31). This showed that the changes of miRNA levels could reflect the process of muscle loss and may be similar to long-term muscle atrophy in DMD. Serum myomiR levels may, therefore, be a biomarker for muscle atrophy, and when combined with clinical data, they may be effective to distinguish DMD from other myopathies.

myomiRs are not only dysregulated in the myopathies with muscle atrophy but also closely related to cardiac disease. Most DMD patients are genetically destined to develop cardiac complications (32). ECG abnormalities can occur before the onset of cardiac dysfunction (12). Our results revealed that patients with cardiac involvement had significantly higher serum levels of miR-1, miR-133a, miR-133b, and miR-499 than non-cardiac-involved patients. These results are not surprising. Both cardiac and skeletal muscles are striated muscles, which are affected by DMD. Except for miR-206, the other six myomiRs, miR-1, miR-133a, miR-133b, miR-208a, miR-208b, and miR-499, can be expressed in cardiac muscles. Moreover, multiple studies have demonstrated that these myomiRs are involved in the development of different types of heart diseases, including dilated cardiomyopathy (33), coronary artery disease (34), cardiac hypertrophy (35, 36), and myocardial fibrosis (37, 38). Although miR-208a and miR-208b were not remarkably higher in the sera of cardiac involvement patients, we believe this may suggest that miR-1, miR-133a, miR-133b, and miR-499 are more sensitive to cardiac involvement. Alternatively, we should include more patients in future, enabling more reliable results.

Genetic testing is currently the gold standard for the diagnosis of DMD. Initially, some small mutations could not be detected by MLPA, so further detection methods such as next-generation sequencing had to be used, which have the disadvantages of long detection time periods and high costs. The use of serum myomiRs to distinguish the common mutation types of DMD would have been extremely useful. However, no significant difference could be observed between serum miRNA levels between the three common types of mutations (deletions, duplications, and small mutations) in this study. In addition, there was no simple relationship between the size of the deletion/duplication and the clinical manifestation of muscular dystrophy. The severity of the disease depended on whether the mutations disrupted the reading frame (39). Therefore, using a more refined grouping method for MD patients may influence the results of future studies. This requires the enrollment of more subjects, especially BMD patients.

We found that serum levels of miR-1, miR-133a, miR-133b, miR-206, miR-208a, miR-208b, and miR-499 were significantly elevated in DMD patients. These miRNAs were positively correlated with lower limb muscle strength and were negatively correlated with muscle functions. Moreover, it has been reported that serum miRNAs returned to normal levels in mdx mice after treatment with an exon skipping strategy (6). Our results suggest that serum muscle-specific miRNAs have the ability to act as novel biomarkers for monitoring pathology and pathophysiology of DMD patients.

## Data Availability Statement

The original contributions presented in the study are included in the article/Supplementary Material, further inquiries can be directed to the corresponding author.

## Ethics Statement

The studies involving human participants were reviewed and approved by the Ethics Committee of the First Affiliated Hospital of Guangxi Medical University. Written informed consent to participate in this study was provided by the participants' legal guardian/next of kin.

## Author Contributions

DL and QM summarized all the information and analyzed the results, and drafted the manuscript together with JZho. QM and JZha performed the experiments. DZ collected the specimens and clinical information. All authors read and approved the final manuscript.

## Funding

This study was supported by the National Natural Science Foundation of China (Grant No. 81760215).

## Conflict of Interest

The authors declare that the research was conducted in the absence of any commercial or financial relationships that could be construed as a potential conflict of interest.

## Publisher's Note

All claims expressed in this article are solely those of the authors and do not necessarily represent those of their affiliated organizations, or those of the publisher, the editors and the reviewers. Any product that may be evaluated in this article, or claim that may be made by its manufacturer, is not guaranteed or endorsed by the publisher.

## Abbreviations

AUC: area under the curve
BMD: Becker muscle dystrophy
CK: creatine kinase
DMD: Duchenne muscular dystrophy
EDL: extensor digitorum longus
ECG: electrocardiography
MLPA: multiplex ligation-dependent probe amplification
MRC: Medical Research Council
NGS: next-generation sequencing
NSAA: North Star Ambulatory Assessment
ROC: receiver operating characteristic
SD: standard deviation
SOL: soleus.
